# Nationwide Monitoring of Antimicrobial-Resistant *Escherichia coli* and *Enterococcus* spp. Isolated From Diseased and Healthy Dogs and Cats in Japan

**DOI:** 10.3389/fvets.2022.916461

**Published:** 2022-06-24

**Authors:** Yukari Furuya, Mari Matsuda, Saki Harada, Mio Kumakawa, Takahiro Shirakawa, Mariko Uchiyama, Ryoko Akama, Manao Ozawa, Michiko Kawanishi, Yoko Shimazaki, Hideto Sekiguchi

**Affiliations:** National Veterinary Assay Laboratory, Ministry of Agriculture, Forestry and Fisheries, Tokyo, Japan

**Keywords:** *Escherichia coli*, *Enterococcus* spp., monitoring, antimicrobial-resistant, companion animals

## Abstract

The Japanese Veterinary Antimicrobial Resistance Monitoring System (JVARM) was established for nationwide monitoring of antimicrobial-resistant bacteria isolated from animals. Here, antimicrobial resistance of *Escherichia coli* and *Enterococcus* spp. isolates from diseased and healthy dogs and cats was investigated. Isolates were collected from diseased dogs and cats and from healthy dogs and cats in 2018 to 2020. Minimum inhibitory concentrations were determined for 1873 *E. coli* and 1383 *Enterococcus* spp. isolates. *E. coli* isolates were most commonly resistant to nalidixic acid [diseased dog (DD), 62.1%; diseased cat (DC), 59.9%; healthy dog (HD), 23.5%; healthy cat (HC, 24.0%] and ampicillin (DD, 54.4%; DC, 64.1%; HD, 28.4%; HC, 25.2%), followed by ciprofloxacin (DD, 45.0%; DC, 44.0%; HD, 12.9%; HC, 10.4%). *Enterococcus* spp. isolates were most resistant to tetracycline (DD, 66.9%; DC, 67.8%; HD, 47.0%; HC, 52.0%), followed by erythromycin (DD, 43.2%; DC, 46.6%; HD, 27.8%; HC, 34.0%) and ciprofloxacin (DD, 27.9%; DC, 43.7%; HD, 9.7%; HC 12.9%). Only a few *E. coli* isolates were resistant to colistin and none were resistant to meropenem. Also, none of the *Enterococcus* spp. isolates we have tested were resistant to vancomycin. The significantly higher resistance rates of *E. coli* and *Enterococcus* spp. isolates from diseased, as opposed to healthy, dogs and cats against most of the tested antimicrobials indicates that the use of antimicrobials could select resistant *E. coli* and *Enterococcus* spp.

## Introduction

The emergence and spread of antimicrobial-resistant (AMR) bacteria is widely recognized as a global health threat (1). The concept of “One Health” is crucial to address this issue, because humans, animals, foods, and environments are potential reservoirs of AMR bacteria and genes (2). In the veterinary field, globally, livestock is the main target for monitoring and risk assessment of AMR bacteria. The Japanese Veterinary Antimicrobial Resistance Monitoring System (JVARM) was established in 1999 to monitor AMR bacteria isolated from livestock but not companion animals (i.e., dogs and cats) as same as many countries (3). In some countries, including Sweden, France and Norway, monitor AMR bacteria in companion animals (4–6).

Although AMR is also major concern in companion animals, there are few monitoring results for risk assessment (7). Studies in Japan detected extended-spectrum beta-lactamase-producing *Escherichia coli* and *bla*_CTX−M_ type β-lactamase genes in samples collected from dogs and cats (8, 9). Additionally, greater rates of enrofloxacin-resistant *Enterococcus* spp. were confirmed in diseased, as opposed to healthy, dogs, and cats (10). These studies revealed potential treatment failure risks in dogs and cats and transmission risks of AMR bacteria from companion animals to humans and vice versa. Therefore, our group developed a national monitoring system for dogs and cats under the JVARM framework to assess the risks according to the strategy of the first national action plan on AMR in Japan (11).

Monitoring of targeted pathogens isolated from diseased dogs and cats was initiated from the veterinary medical perspective. In addition, as a fundamental data of AMR bacteria, *E. coli* and *Enterococcus* spp. from healthy animals is also important to monitor, since *E. coli* and *Enterococcus* spp. are globally known as a multi-sectoral indicator bacteria and often selected as target bacteria of AMR monitoring. Also, *E. coli* and *Enterococcus* spp. are commensal microorganisms causing opportunistic infections (12, 13). Therefore, we collected *E. coli* and *Enterococcus* spp. from both diseased and healthy dogs and cats for this monitoring.

Monitoring of both diseased and healthy dogs and cats is almost unprecedented; however, the aim of this study was to summarize the results of nationwide monitoring of AMR *E. coli* and *Enterococcus* spp. from diseased and healthy dogs and cats in Japan.

## Materials and Methods

### Sampling

In order to determine an appropriate number of samples to avoid bias, all prefectures of Japan were divided into blocks and the numbers of samples were calculated based on the number of small animal clinics in each block. Hence, collection of only one *E. coli* and/or *Enterococcus* spp. isolate from each clinic, which was to be in total 200 isolates, suggest that each bacterial species should be collected from dogs and cats per year.

*E. coli* strains were isolated from clinical urine or genital tract samples and *Enterococcus* spp. strains were isolated from clinical urine or ear samples of diseased dogs and cats. All of *E. coli* and *Enterococcus* spp. isolated from diseased dogs and cats are isolated and identified in clinical laboratories from clinical samples.

In addition, *E. coli* and *Enterococcus* spp. were isolated from rectal swabs of healthy dogs and cats brought to small animal clinics for either medical checkups or vaccinations, but not treatment.

### Informed Consent for Sampling

Clinical samples from diseased dogs and cats, submitted to clinical laboratories, were sourced from veterinarians and owners and used under the agreement of the use for research.

The owners were explained the purpose of the surveillance and requirement of isolates from healthy dogs and cats, and written informed consent was obtained prior to sample collection.

All of the isolates were anonymized.

### Identification of *E. coli* and *Enterococcus* spp.

For diseased dogs and cats, clinical laboratories cultured suspected *E*. *coli* isolates on MacConkey agar or deoxycholate-hydrogen sulfide-lactose (DHL) agar and identified through the IMViC test, which is a combination of the indole, methyl red or Voges–Proskauer and citrate tests or using matrix assisted laser desorptionization-time of flight mass spectrophotometry (MALDI TOF MS) (Bruker Daltonics, Germany) or MicroScanWalkAway Plus System (Beckman Coulter, Inc., Japan) for automated identification. Suspected *Enterococcus* spp. were cultured on Trypticase soy agar with 5% sheep blood or phenylethyl alcohol sheep blood agar and identified using the catalase test and confirmed colonies on EF agar (Nissui Pharmaceutical Co., Ltd., Japan) or used MicroScanWalkAway Plus System or MALDI TOF MS. We confirmed *E. coli* by colored colonies on DHL agar, and *Enterococcus* spp. using the Rapid ID32 Strep kit (BioMerieux Vitek, Marcy-I'Etoile, France) and the oxidase test.

For healthy dogs and cats, suspected *E. coli* isolates were tested using DHL agar, then cultured on triple sugar iron agar, and subjected to the IMViC test. Suspected *Enterococcus* spp. isolates were smeared on enterococcosel agar and three colonies were randomly sampled and subjected to Gram staining, the catalase test and the pyrrolidonyl arylamidase test, and then cultured in heart infusion broth with NaCl. The species of the suspected *Enterococcus* spp. isolates were identified using the Rapid ID32 Strep kit.

### Antimicrobial Susceptibility Testing

The tested antimicrobials were selected from two perspectives, in order to enable comparison with existing data of livestock in JVARM and to grasp resistant rates of antimicrobials frequently used in small animal clinical practices.

The *E. coli* isolates were tested for minimum inhibitory concentrations (MICs) of ampicillin, cefazolin, cephalexin, cefotaxime, meropenem, kanamycin, gentamicin, streptomycin, tetracycline, chloramphenicol, colistin, ciprofloxacin, nalidixic acid, and trimethoprim/sulfamethoxazole.

The *Enterococcus* spp. isolates were tested for MICs of ampicillin, gentamicin, erythromycin, azithromycin, ciprofloxacin, chloramphenicol, and tetracycline. MICs of vancomycin was assessed for isolates from diseased dogs and cats collected in 2019 and 2020, and all isolates from healthy dogs and cats.

MICs were calculated using a standardized microdilution method in accordance with the Clinical and Laboratory Standards Institute (CLSI) standard (14) using “Dry Plate ‘Eiken' 192” bacterial drug sensitivity testing reagent (EIKEN Chemical Co., Ltd., Japan). The breakpoints as listed in CLSI document M100 (15) and VET01S (16) were applied. Considering the purpose of nationwide continual monitoring, break points set in M100 were adopted instead of break points set by each samples' origins in VET01S. *Escherichia coli* ATCC 25922, *Pseudomonas aeruginosa* ATCC 27853, and *Enterococcus faecalis* ATCC29212 were used as control strains.

### Statistics Analysis

The Fisher's exact test was used to identify differences in the resistance rates among diseased and healthy dogs and cats. A probability *p* < 0.05 was considered statistically significant.

## Results

In total, 1,873 *E. coli* and 1,383 *Enterococcus* spp. isolates were collected. Eight hundred ninety one *E. coli* isolates were collected from diseased dogs and cats and 982 from healthy dogs and cats, whereas 695 *Enterococcus* spp. isolates were collected from diseased dogs and cats and 688 from healthy dogs and cats. All of the isolates were collected in 2018, 2019, and 2020.

For *Enterococcus* spp. isolates, *E. faecalis* [diseased dog (DD), 74.7% (287/384); diseased cat (DC), 62.7% (195/311); healthy dog (HD), 74.5% (322/432); healthy cat (HC), 79.7% (204/256)], *E. faecium* [DD, 17.4% (67/384); DC, 29.3% (91/311); HD, 8.3% (36/432); HC, 4.3% (11/256)], *E. gallinarum* [DD, 2.9% (11/384); DC, 2.9% (9/311); HD, 6.0% (26/432); HC, 3.9% (10/256)], *E. casseliflavus* [DD, 1.0% (4/384); DC, 1.3% (4/311); HD, 2.3% (10/432); HC, 1.2% (3/256)], *E. hirae* [DD, 0.3% (1/384); DC, 0.6% (2/311); HD, 3.5% (15/432); HC, 3.5% (9/256)], *E. avium* [DD, 2.1% (8/384); DC, 1.0% (311); HD, 1.6% (7/432); HC, 3.9% (10/256)], *E. durans* [DD, 0.8% (3/384); DC, 0.6% (2/311); HD, 3.5% (15/432); HC, 3.1% (8/256)] and other *Enterococcus* spp. isolates were detected.

The MIC profiles of the *E. coli* isolates are shown in Table 1. More than 30% of the isolates from both diseased dogs and cats were resistant to nalidixic acid (DD, 62.1%; DC, 59.9%), ampicillin (DD, 54.4%; DC, 64.1%), ciprofloxacin (DD, 45.0%; DC, 44.0%), cefazolin (DD, 34.8%; DC, 36.4%), cephalexin (DD, 35.4%; DC, 37.8%) and cefotaxime (DD, 31.2%; DC, 31.7%). In contrast, <30% of the isolates from healthy dogs and cats were resistant to all antimicrobials. Resistance to ampicillin was most common (HD, 28.4%; HC, 25.2%), followed by nalidixic acid (HD, 23.5%; HC, 24.0%) and cefazolin (HD, 15.1%; HC, 12.2%). None of the *E. coli* isolates were resistant to meropenem and few were resistant to colistin (DD, 0.0%; HD, 0.4%; DC, 0.6%; HC, 0.2%). With the exception of meropenem and colistin, the resistance rates to all antimicrobials were significantly (*p* < 0.01) greater among the isolates from diseased, as opposed to healthy, dogs and cats.

**Table 1:** MIC for *E. coli* isolated from diseased and healthy dogs and cats in Japan.

**Antimicobial agent**			**Diseased dog (*****n*** **=** **509)**			**Healthy dog (*****n*** **=** **490)**			**Diseased cat (*****n*** **=** **382)**			**Healthy cat (*****n*** **=** **492)**		
	**Range**	**Breakpoint**	**MIC_**50**_**	**MIC_**90**_**	**Number of resistant isolates (%)**	**MIC_**50**_**	**MIC_**90**_**	**Number of resistant isolates (%)^**a**^**	**MIC_**50**_**	**MIC_**90**_**	**Number of resistant isolates (%)**	**MIC_**50**_**	**MIC_**90**_**	**Number of resistant isolates (%)^**a**^**
	**(mg/L)**	**(mg/L)**	**(mg/L)**	**(mg/L)**		**(mg/L)**	**(mg/L)**		**(mg/L)**	**(mg/L)**		**(mg/L)**	**(mg/L)**	
Ampicillin	≤ 4–>128	32	>128	>128	277 (54.4%)	8	>128	139 (28.4%)**	16	>128	229 (64.1%)	≤ 4	>128	124 (25.2%)**
Cefazolin	≤ 2–>128	32	≤ 2	>128	177 (34.8%)	≤ 2	>128	74 (15.1%)**	≤ 2	>128	130 (36.4%)	≤ 2	64	60 (12.2%)**
Cefalexin	≤ 2–>128	32	8	>128	180 (35.4%)	8	>128	74 (15.1%)**	8	>128	135 (37.8%)	8	>128	67 (13.6%)**
Cefotaxime	≤ 0.5–>64	4	≤ 0.5	64	159 (31.2%)	≤ 0.5	8	56 (11.4%)**	≤ 0.5	64	113 (31.7%)	≤ 0.5	≤ 0.5	33 (6.7%)**
Meropenem	≤ 0.5–>8	4	≤ 0.5	≤ 0.5	0 (0.0%)	≤ 0.5	≤ 0.5	0 (0.0%)	≤ 0.5	≤ 0.5	0 (0.0%)	≤ 0.5	≤ 0.5	0 (0.0%)
Streptomycin	≤ 4–>128	–	8	>128	–	8	128	–	8	>128	–	≤ 4	32	–
Gentamicin	≤ 2–>64	8	≤ 2	32	77 (15.1%)	≤ 2	≤ 2	22 (4.5%)**	≤ 2	16	46 (12.9%)	≤ 2	≤ 2	18 (3.7%)**
Kanamycin	≤ 4–>128	64	≤ 4	16	31 (6.1%)	≤ 4	8	21 (4.3%)**	≤ 4	16	27 (7.6%)	≤ 4	≤ 4	14 (2.8%)**
Tetracycline	≤ 2–>64	16	4	>64	122 (24.0%)	≤ 2	64	68 (13.9%)**	≤ 2	>64	88 (24.6%)	≤ 2	4	48 (9.8%)**
Chloramphenicol	≤ 4–>128	32	8	32	60 (11.8%)	8	16	26 (5.3%)**	8	16	32 (9.0%)	8	8	11 (2.2%)**
Colistin	≤ 0.5–>16	4	≤ 0.5	≤ 0.5	0 (0.0%)	≤ 0.5	≤ 0.5	2 (0.4%)	≤ 0.5	≤ 0.5	2 (0.6%)	≤ 0.5	≤ 0.5	1 (0.2%)
Nalidixic acid	≤ 4–>128	32	>128	>128	316 (62.1%)	≤ 4	>128	115 (23.5%)**	>128	>128	214 (59.9%)	≤ 4	>128	118 (24.0%)**
Ciprofloxacin	≤ 0.06–>8	1	0.5	>8	229 (45.0%)	≤ 0.06	8	63 (12.9%)**	0.25	>8	157 (44.0%)	≤ 0.06	1	51 (10.4%)**
Sulfamethoxazole/ trimethoprim	≤ 9.5/0.5–>152/8	76/4	≤ 9.5/0.5	>152/8	108 (21.2%)	≤ 9.5/0.5	>152/8	52 (10.6%)**	≤ 9.5/0.5	>152/8	84 (22.0%)	≤ 9.5/0.5	≤ 9.5/0.5	45 (9.1%)**

^a^p-values were determined by Fisher's exact test. **p < 0.01.

The MICs of the *Enterococcus* spp. isolates are shown in Table 2. The isolates from diseased dogs and cats were most commonly resistant to tetracycline (DD, 66.9%; DC, 67.8%), followed by erythromycin (DD, 43.2%; DC, 46.6%) and ciprofloxacin (DD, 27.9%; DC, 43.7%). The isolates from healthy dogs and cats were also highly resistant to tetracycline (HD, 47.0%; HC, 52.0%), erythromycin (HD, 27.8%; HC, 34.0%) and ciprofloxacin (HD, 9.7%; HC, 12.9%). Isolates from diseased dogs and cats showed significantly higher resistance rates (*p* < 0.01) to all antimicrobials, except for chloramphenicol and vancomycin, than those from healthy dogs and cats.

**Table 2:** MIC for *Enterococcus* spp. isolated from diseased and healthy dogs and cats in Japan.

**Antimicobial agent**			**Diseased dog (*****n*** **=** **384/306**^**b**^**)**				**Healthy dog (*****n*** **=** **432)**			**Diseased cat (*****n*** **=** **311/251**^**b**^**)**			**Healthy cat (*****n*** **=** **256)**		
	**Range**	**Breakpoint**	**Number of samples**	**MIC_**50**_**	**MIC_**90**_**	**Number of resistant isolates (%)**	**MIC_**50**_**	**MIC_**90**_**	**Number of resistant isolates (%)^**a**^**	**MIC_**50**_**	**MIC_**90**_**	**Number of resistant isolates (%)**	**MIC_**50**_**	**MIC_**90**_**	**Number of resistant isolates (%)^**a**^**
	**(mg/L)**	**(mg/L)**		**(mg/L)**	**(mg/L)**		**(mg/L)**	**(mg/L)**		**(mg/L)**	**(mg/L)**		**(mg/L)**	**(mg/L)**	
Ampicillin	≤ 0.5–>64	16	384	1	>64	68 (17.7%)	1	2	20 (4.6%)**	1	>64	94 (30.2%)	1	2	6 (2.3%)**
Vancomycin	≤ 0.12–>32	32	306^b^	1	2	0 (0.0%)	1	2	0 (0.0%)	1	2	0 (0.0%)	1	2	0 (0.0%)
Gentamicin	≤ 1–>64	–	384	8	>64	–	8	64	–	8	>64	–	8	>64	–
Erythromycin	≤ 0.25–>32	8	384	2	>32	166 (43.2%)	2	>32	120 (27.8%)**	4	>32	145 (46.6%)	2	>32	87 (34.0%)*
Azithromycin	≤ 0.25–>32	–	384	8	>32	–	4	>32	–	8	>32	–	4	>32	–
Ciprofloxacin	≤ 0.25–>32	4	384	1	>32	107 (27.9%)	1	2	42 (9.7%)**	2	>32	136 (43.7%)	1	16	33 (12.9%)**
Chloramphenicol	≤ 1–>64	32	384	8	64	61 (15.9%)	8	32	52 (12.0%)	8	64	45 (14.5%)	8	32	34 (13.3%)
Tetracycline	≤ 0.5–>64	16	384	64	>64	257 (66.9%)	1	64	203 (47.0%)**	64	64	211 (67.8%)	32	64	133 (52.0%)**

^a^p-values were determined by Fisher's exact test. *p < 0.05; **p < 0.01.

^b^*MIC for Vancomycin was tested for all isolates except diseased dogs and cats in 2018*.

Notably, 91.0% (61/67) and 92.1% (82/89) of the *E*. *faecium* from diseased dogs and cats, respectively, 0.3% (1/287) and none (0/195) of the *E*. *faecalis* isolates from diseased dogs and cats, respectively, were resistant to ampicillin, while 22.2% (8/36) and 9.1% (1/11) of the *E*. *faecium* and 0.3% (1/322) and none (0/204) of *E*. *faecalis* isolates from healthy dogs and cats were resistant to ampicillin. Even by each years, *E*. *faecium* [DD, 100% (15/15), 90.0% (27/30), 86.4% (19/22); DC, 100% (18/18), 94.3% (33/35), 81.6% (31/38); HD, 29.2% (7/24), 0.0% (0/3), 0.0% (0/9); HC, 14.3% (1/7), 0.0% (0/1), 0.0% (0/3), in 2018, in 2019, and in 2020, respectively] and *E*. *faecalis* [DD, 0.0% (0/52), 0.0% (0/100), 0.8% (1/130); DC, 0.0% (0/39), 0.0% (0/62), 0.0% (0/94); HD, 1.0% (1/100), 0.0% (0/123), 1.0% (1/9); HC, 14.3% (1/7), 0.0% (0/1), 0.0% (0/3), in 2018, in 2019, and in 2020, respectively], showed pattern of certain resistant rates to ampicillin.

**Figure 1:**
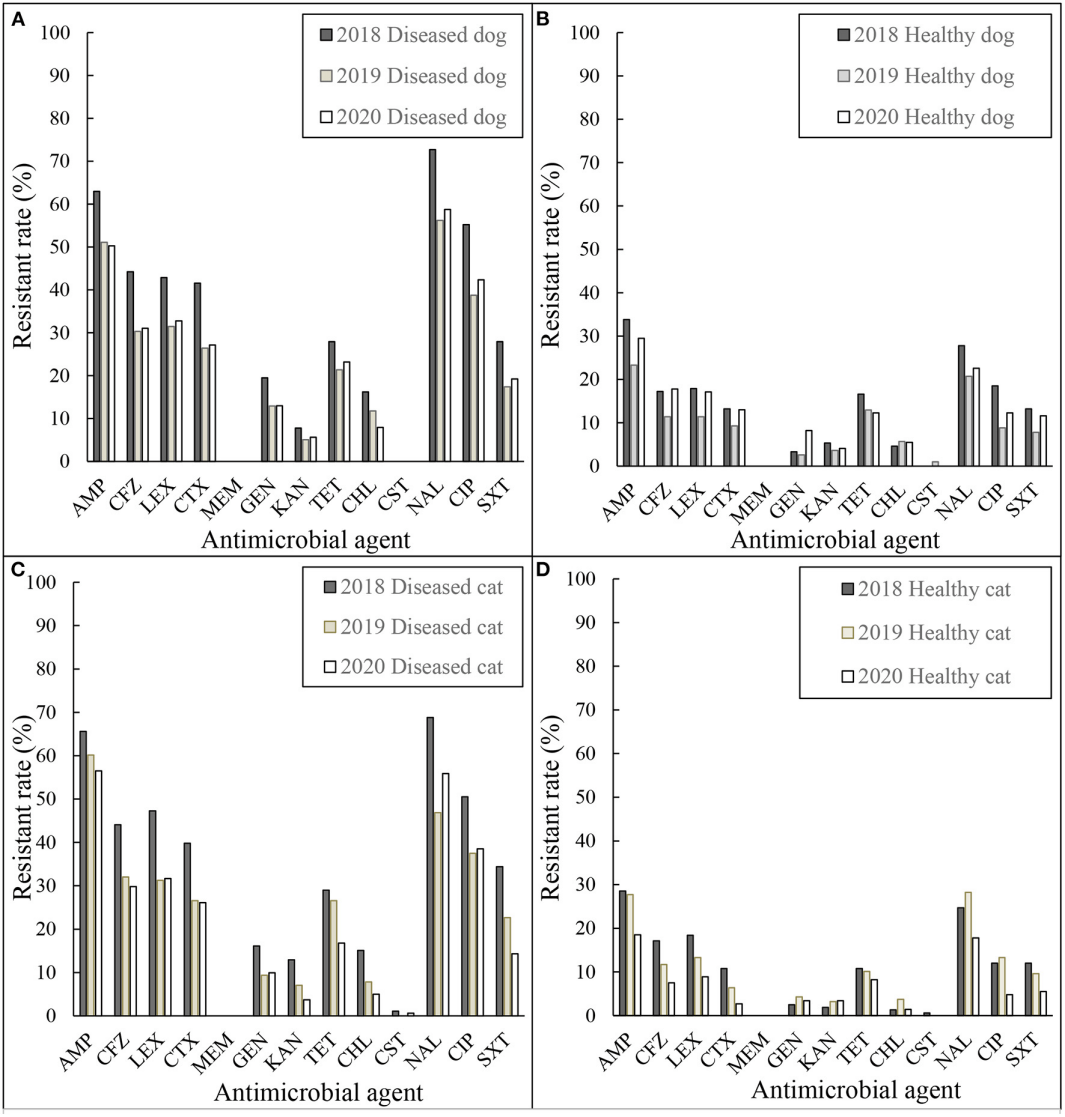
Resistance rates of *E. coli* **(A)** diseased dog 2018–2020, **(B)** healthy dog 2018–2020, **(C)** diseased cat 2018–2020, and **(D)** healthy cat 2018–2020. AMP, ampicillin; CFZ, cefazolin; LEX, cephalexin; CTX, cefotaxime; MEM, meropenem; STR, streptomycin; GEN, gentamicin; KAN, kanamycin; TET, tetracycline; CHL, chloramphenicol; CST, colistin; NAL, nalidixic acid; CIP, ciprofloxacin; SXT, sulfamethoxazole/trimethoprim.

**Figure 2:**
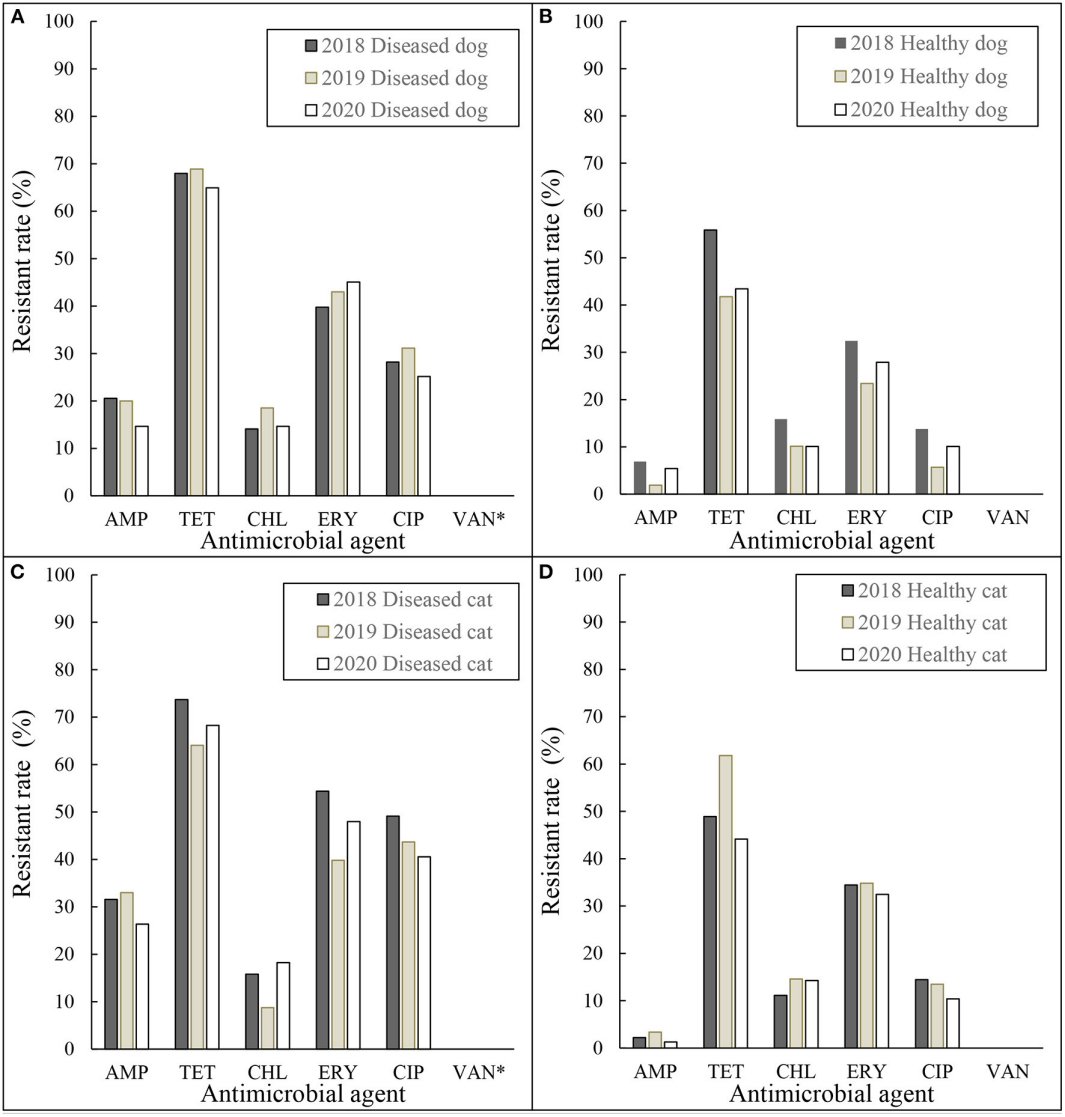
Resistance rates of *Enterococcus* spp. **(A)** diseased dog 2018–2020, **(B)** healthy dog 2018–2020, **(C)** diseased cat 2018–2020, and **(D)** healthy cat 2018–2020. AMP, ampicillin; TET, tetracycline; CHL, chloramphenicol; ERY, erythromycin; CIP, ciprofloxacin; VAN, vancomycin. *MIC for Vancomycin was tested for all isolates except diseased dogs and cats in 2018.

The resistance rates of the *E. coli* and *Enterococcus* spp. isolates grouped by year are shown in Figures 1, 2. In Figure 1, the resistant rates of *E. coli* isolates collected from diseased dogs and cats in 2018 were higher than those collected in 2019 and 2020, whereas the resistant rates of *E. coli* isolates from healthy dogs and cats collected in 2018 and 2019 were similar.

## Discussion

Overall, resistance to most of the tested antimicrobials was significantly higher in diseased, as opposed to healthy, dogs and cats. In this study, background information of the samples to trace antimicrobial use was limited; however, diseased dogs and cats are more likely to have been treated with antimicrobials. According to Nippon AMR One Health Report (17), the highest volume of veterinary antimicrobials estimated sales for dogs and cats were cephalosporins, especially the first generation cephalosporins followed by penicillins. Conversely, almost none carbapenems were sold. It is may be correlated with resistant rates of *E. coli* isolated from diseased dogs and cats against cefazolin, cefalexin, cefotaxime, and ampicillin, which were more than 30%, and none were resistant to meropenem. This results indicate that among diseased dogs and cats that were administered antimicrobials, the use of antimicrobials is may be responsible for the selective pressure of bacterial flora of *E. coli* and *Enterococcus* spp. In addition to the fact that there were differences in resistance rates among diseased and healthy dogs and cats, there were similarities in the types of antimicrobials they showed resistance to (e.g., *E. coli* resistance to nalidixic acid and ampicillin; *Enterococcus* spp. resistance to tetracycline, erythromycin, and ciprofloxacin).

According to data collected by JVARM in 2016 and 2017 (18), *E. coli* isolates from cattle, pigs and broilers in slaughterhouses were most resistant to streptomycin (19.0–51.3%) and tetracycline (21.0–56.7%), while isolates from diseased livestock were highly resistant to tetracycline (54.5–87.3%) followed by streptomycin (38.9–74.5%), ampicillin (33.3–74.5%) and chloramphenicol (11.1–69.6%). Resistance to cefotaxime and ciprofloxacin was generally lower in *E*. *coli* isolates from healthy than diseased livestock (0.0–5.7 and 2.9–8.9 vs. 0.0–12.0 and 11.1–28.5%, respectively). Notably, <30% of all *E*. *coli* isolates from diseased and healthy cattle, pigs and broilers, but >30% of those from diseased dogs and cats were resistant to cefotaxime and ciprofloxacin. In contrast, *E. faecalis* and *E. faecium* isolates collected from healthy pigs and broilers in 2017 were highly resistant to oxytetracycline (31.8–84.6%) and erythromycin (27.3–61.5%) (18). Kimura et al. (19), also found similar trends for *Enterococcus* spp. isolated from diseased companion animals in Japan (non-susceptibility rates against doxycycline and minocycline of 40–56% and erythromycin of 40–93%).

Data collected by the Japan Nosocomial Infections Surveillance System (20) indicate that trends in *E. coli* in human resistance to penicillins, quinolones/fluoroquinolones, and cephalosporins are similar to those of dogs and cats, in addition to high resistance of *Enterococcus* spp. to tetracycline and erythromycin.

Although the trends in resistance of *Enterococcus* spp. concurred with those of all animal species considered in this study, there were notable differences in the AMR profiles of *E. coli* isolates from humans, dogs and cats vs. livestock, which may be due to differences in the antimicrobial classes used for treatment of infections.

Consistent with previous studies, most *E. faecium* and few *E*. *faecalis* isolates from diseased dogs and cats were resistant to ampicillin (21). However, interestingly, the resistance rates of *E. faecium* from healthy dogs and cats were significantly lower than from diseased dogs and cats (22.2 and 9.1 vs. 91.0 and 92.1%, respectively), even in each years. Jackson et al. reported that 47.4 and 51.6% of *E. faecium* isolates from healthy dogs and cats in the United States were resistant to penicillin (22), which are not exceptionally high resistance rates. Origins of the human *E*. *faecium* infection are broadly categorized as hospital or community-associated (23, 24). Most cases of hospital-associated *E. faecium* are resistant to ampicillin while community-associated cases are generally susceptible to ampicillin, suggesting the possibility of similar types of *E. faecium* infection of dogs and cats.

As shown in Figures 1, 2, there were small annual fluctuations with regular, rather than random patterns. Despite collecting only 3 years of data, this monitoring system seemed to be useful to illustrate trends. Although there were differences in the resistant rates between diseased and healthy, resistant rates between animal species, dogs and cats, were similar in both *E. coli* and *Enterococcus* spp. isolates.

Few countries monitor AMR bacteria for dogs and cats. Swedish Veterinary Antimicrobial Resistance Monitoring (SVARM) program (4), refers to MICs of *E. coli* from clinical urine samples of dogs and cats. In the SVARM report, 13% of *E. coli* isolates from dogs and 16% from cats were resistant to ampicillin. In France, the national surveillance network for antimicrobial resistance in bacteria from diseased animals (RESAPATH) (5), collects data *via* the disc diffusion method of *E. coli* isolates from clinical samples of dogs and cats with various pathologies. According to the RESAPATH data, 70, 61, and 73% of *E. coli* isolates from kidney/urinary tract, skin/soft tissue, and otitis samples, respectively, from dogs and 70% from cats with all pathologies were susceptible to amoxicillin. Furthermore, according to the national monitoring program data for antimicrobial resistance in the veterinary and food production sectors in Norway (NORM-VET) (6), 20.2 and 46.5% of *E*. *coli* isolates from urine of dogs with urinary tract infections and other infections, respectively, were resistant to ampicillin. It should be noted that the SVARM data includes ECOFFs as defined by the EUCAST, while the RESAPATH system uses the NF U47-107 standard of the Antibiogram Committee of the French Society of Microbiology, and the NORM-VET program uses the clinical breakpoints or ECOFFs.

Each of the cited surveillance systems adopted different breakpoints and there were differences in the numbers and types of tested antimicrobial agents as well as the sampling methods (passive sample collection method of the SVARM, RESAPATH and NORM-VET systems vs. an active sample collection method in the present study). Therefore, data comparisons were challenging. In our study, 54.4 and 64.1% of the *E. coli* isolates from diseased dogs and cats, respectively, were resistant to ampicillin, which were higher rates than reported in Sweden, France and Norway, where aggressive measures against AMR have been enacted.

In the present study, the resistance rates of *E. coli* and *Enterococcus* spp. isolates from diseased dogs and cats were significantly higher than those from healthy dogs and cats against most of the tested antimicrobials, indicating that use of antimicrobials could be selective pressure for resistant *E. coli* and *Enterococcus* spp.

The limitation of our study lies on difference of sample origins between diseased and healthy dogs and cats, urine/genital/ear sample origin vs. rectal swab origin due to collect enough numbers of strains. Also, there are lack of genetic data including serotype. However, our results are valuable to know AMR situation in dogs and cats in Japan and useful to consider AMR measures.

In conclusion, this is the huge step toward continued AMR monitoring of isolates from diseased and healthy dogs and cats. To the best of our knowledge, nationwide monitoring systems of AMR bacteria isolated from both diseased and healthy dogs and cats are rare. In 2020 (25), our group published guidelines for use of antimicrobials for companion animal veterinarians to avoid selection of AMR bacteria. The results of this study suggest that it is crucial to promote prudent use of antimicrobials in companion animals and to continue monitoring trends in AMR bacteria.

## Data Availability Statement

The raw data supporting the conclusions of this article will be made available by the authors, without undue reservation.

## Author Contributions

YF, HS, YS, RA, MO, MM, SH, and MKa contributed to conception and design of the study. MU, MKu, and TS organized the sampling methods and approached to the related organizations. All authors contributed to manuscript revision, read, and approved the submitted version.

## Conflict of Interest

The authors declare that the research was conducted in the absence of any commercial or financial relationships that could be construed as a potential conflict of interest.

## Publisher's Note

All claims expressed in this article are solely those of the authors and do not necessarily represent those of their affiliated organizations, or those of the publisher, the editors and the reviewers. Any product that may be evaluated in this article, or claim that may be made by its manufacturer, is not guaranteed or endorsed by the publisher.
